# Friendly Hints to a Reviewer

**Published:** 1850-09

**Authors:** Samuel Jackson

**Affiliations:** Formerly of Northumberland


*Friendly hints to a Reviewer.*—Something more on moist hot air.
By Samuel Jackson, M. D., (formerly of Northumberland.)

To the Editor of the Medical Examiner.

You published my hints on wetting the streets, in the June No. of the Examiner; you have seen the review of it by Th. Bell, M. D., in the July No. of the Western Journal of Med. and Surg., of which he is a co-editor. He has in spirit if not in sentiment arraigned me as unphilosophical, confused in mind,* and as justly suspected of "murmuring at Providence." I am very unwilling to answer the reviewer, and I therefore send you only a rambling commentary, for it has not been consistent with either my leisure or taste to spend much time on the subject. I am sorry that I did not see the review till it was handed me by our friend Dr. Isaac

* "It seems to us that Dr. Jackson set about his investigations with a confused set of ideas."

Parrish, the 26th ult., when your last No. wherein this reply ought to have appeared, had gone through the press.

As I do not feel that I can safely spare the least portion of my little reputation for the character of *Religious*, among a people whom M. de Tocqueville considers as more *religiously* persecuting than any nation of Europe, I shall first defend myself in this vulnerable quarter. Some physicians have had the fame of making religion a rail-road to the paradise of a profitable practice; but, alas for me, poor sinner, I am like Swift, "a hypocrite reversed," for I am openly running backward on the same track.

I have wickedly said—"The washing of the pavements is a very great evil whether in winter or in summer, independently of the dampness it adds to the atmosphere. If done in the morning, it annoys passangers by wetting their feet, and not unfrequently the pavements become thereby covered with ice and dangerous to all pedestrians: if done in the evening as it often is, it is sure to have this effect in freezing weather; and in the milder, they remain wet till the next day noon. Now it is a fact, that there are many sick people who cannot take fresh air except by walking, and to them, and indeed to all delicate people, the damp pavements present a very formidable obstacle. This is not a hypothesis of mine, but a stubborn fact from the lips and hearts of many sickly people."

Now, says the Reviewer, "this sounds to us very much like what some people call 'murmuring at Providence.' He who orders all things, has ordained rains for a Philadelphia winter, and without any regard to 'sick people who cannot take fresh air, except by walking,' or to all delicate people, or to Dr. Jackson's opinion that it is a great evil, Providence has ordained freezing weather for Philadelphia, and we cannot see how Dr. Jackson's strong objections to rain and frozen pavements can alter the movements of Heaven. His remarks apply as forcibly against winter rains as against the use of Schuylkill water. According to Dr. Jackson's hypothesis it is wrong to send rain on a winter's evening, because if the weather is cold the pavements will freeze, and if not cold enough for that, 'they will remain wet until next day noon.' Dr. Jackson evidently thinks there should be no rain in freezing weather; and that all winter rains should fall in the morning, and Providence should send out a drying sun, to dry up the pavements sooner than next day at noon. Really we cannot see how Dr. Jackson is to be accommodated. Even if the city authorities of Philadelphia could be persuaded to bring water 'from the Lehigh, or the Delaware far above the Falls,' the evil would not be removed, for Providence would prove to be rather an untractable subject for reform on the puny reasonings of an erring mortal."

Observe, kind reader, he says—"this sounds to us very much like what some people call—murmuring at Providence:" not merely *much* but *very* much. I will not say that his replication sounds *very much* as though written by St. Dominic's Chief Inquisitor. Such a bill of indictment, seems to require a formal recantation and open repentance, if it be founded in truth. I have placed the crime and the bill side by side as he has quoted and written them, in addition to which a few words will suffice. God has created and governed the world in benevolence and wisdom; he sends rain, frost, lightning, and storms so as to effect the greatest possible general good; yet no man was ever accused of murmuring because he turned aside the fiery bolts, or invoked or deprecated rain, as our ministers are ordered to do from the pulpit, *pro re. nata.* No one ever heard Mr. Espy called impious because, he would bring rain into a particular region though he would thus withdraw it from lands where Providence had sent his clouds. Have men not made changes in the earth without this murmuring—levelled mountains, filled up marshes, dug canals, turned aside rivers; and have not the people of Louisville built a huge bridge which the law will soon compel them to unbuild? (This however is not so much a murmuring as an open rebellion, for the New Testament enjoins all men to obey the laws.) Heaven has given the Reviewer hair and nails and covered his body with a tough hide—why does he not in his anti-murmuring zeal, leave these all to nature and go about like Nebuchadnezzar, with a naked body, "birds claws, and eagles feathers?"

Heaven has sent fevers into many parts of the earth—would any court be accused of this "murmuring" if they should forbid the erection of mill dams or the turning aside of rivers by which the country would be filled with vapor, malaria thereby rendered more active, and fevers multiplied? Has not Providence sent us cases of small pox the last 50 years, and are we therefore justified in propagating the disease, or would not the law interfere? Impious law, which would thus prevent what is so clearly appointed by the example of heaven itself!! Men are often observing to each other that the pavements are too wet or too icy for walking with safety and comfort, this they do without this "murmuring"—would they not be justifiable in preventing their busy-body neighbors from making them wet or icy at times when Heaven had left them dry and safe?

But the Reviewer *constructs* another accusation—" according to Dr. J.'s hypothesis, it is wrong to send rain on a winter's evening, because if the weather is cold, the pavements will freeze," &c. If *constructive* treason against the state is not allowable in secular courts, I shall hope it cannot be entertained even in the ecclesiastical court of public opinion, so that we shall come off unscathed and be able to whistle down the Reviewer with the good old tune,

> Où courrez-vous donc Monsieur l' abbé ?

> Vous allez vous casser le nez.

> O Doctor, Doctor, where do you run

> In haste to break your nosey ?

Let the Reviewer remember the *Tale of a Tub* and profit by Jack's intemperate zeal for religion and his broken nose.

The Reviewer says:—

"That the sprinkling of water, on streets paved with stone cannot have a baleful influence is manifest from a great variety of facts. Before the streets of Louisville were paved, the rains of summer caused a great mortality. This was not on account of the mere humidity of the atmosphere, for the rains that fall in summer now, make the air as humid as that of 1822, but without any evil effect. All parts of the city of Louisville are equally sprinkled by rains, but all parts are not alike unhealthy. A considerable portion of the city retains its health, amidst the heats of summer, the blasts of autumn, the sleets of winter, and the rains of spring, and it is, therefore, evident, that we must look for something else besides the mere dryness or humidity of the atmosphere, as a cause of disease. The drenching rains of summer that fell for a month, in Lexington, Kentucky, in 1833, and caused a frightful cholera epidemic, did not affect all parts of that city alike. The ravages of the epidemic radiated from an unpaved portion of the city, but large squares in Lexington were exempt from the epidemic disease. But the exempted, equally with the afflicted portions had a humid atmosphere. That portion of the city of Cincinnati that is most constantly sprinkled with water from the hydrants is very far from being the most sickly part of the city. And that portion of Louisville most constantly sprinkled by water-carts is precisely the most salubrious part."

I cannot understand this passage unless the Reviewer means by *disease* the koinomiasmatic fevers, for he says,—" a considerable part of the city retains its health." This must be a cant phrase for an exemption from the fevers of the Western states, for surely there must have been sickness of various kinds in every part of the city. Let us proceed then on this assumption. Before the streets of Louisville were paved, they were no doubt covered for a long time after heavy rains, by mudholes, as thick as they are found in the unpaved roads in the vicinity of every city; now to say that no more vapour arises from such streets than from those that are well paved and therefore quickly dried, is a glaring absurdity. This is the ground which, *in the confined air of a city*, affords the most vapor. The earth is heated, the water is spread abroad in shallows and mixed with earth over hundreds of acres—what more is wanting to produce steam ?

But says the Reviewer, " we must look for something else besides the mere dryness or humidity of the atmosphere as a cause of disease." Undoubtedly you must if you have fevers, but with this unknown something and with fevers, I told the Reviewer in my preface that my reasoning had nothing to do, only so far as they were rendered worse by an atmosphere too moist. But mark " the confused ideas" of which he blames me : he has just said " we must look for something besides mere dryness and humidity as a cause of disease" and yet the very next words are these— " the drenching rains that fell for a month in Lexington in 1833, caused a frightful cholera epidemic." Ἑυρηκαμεν ἑυρηκαμεν συγχαιρωμεν we have found him, we have found him, let us rejoice together, said the priests of Osiris—let physicians congratulate with each other that the cause of cholera has been at last discovered ; it is nothing but a " drenching rain."

He says the disease " radiated from an unpaved portion of the city :" it no doubt began there first, for there were the mudholes and the consequent vapors that gave activity to the mysterious poison, and had already broken down the strength, the *vivida vis*, of the people, thus rendering them more susceptble. Oh, no, no, says the Reviever, " the exempted equally with the afflicted portions, had a humid atmosphere." Did the Reviewer verify this incredible assertion by the hygrometer ? Is it consistent with experience particularly in London, that the muddy parts of a city are as free from vapor as the paved and dry ? But the portions of Louisville and Cincinnati that are sprinkled, are not the most unhealthy, says the Reviewer. Very likely, because in these portions live no doubt the very people who are least exposed to sickness, whose streets are paved and dry with the exception of this sprinkling—and mark, I no where asserted that sprinkling produced disease ; I only said that it would, if made general, contribute, with the washing of pavements, side-yards, back-yards, and alleys, to the hygrometric state of the atmosphere; and I left it to the Society to which it was addressed, to argue, whether it could be in sufficient quantities to injure the public health.

As to its producing any specific disease, as malarious fever or cholera, this idea never entered my head. I have to confess however that I used one unguarded expression or word—" this impure water is showered over the streets where mingling with the *miasma-gennetic* materials there spread abroad, a poisonous exhalation is diffused thro' the air." But here the intelligent reader will see clearly that by the word *miasma* I did not mean the malaria of fevers; I took the word in its primitive and general sense from μιαινω to pollute, defile &c. So we may say the air within and around the cemetery *Des Innocens* was polluted, breaking down the health of the vicinage, producing debility, incurable headache, and nervous tremors, but then there was no specific fever generated thereby. Malarious fevers did not come into my argument, for they are scarcely thought of in the interior of Philadelphia.

When I relate from Park, Lind, and others, that different parts of the African torrid zone are healthy during the dry season, I am not crazy enough to suppose there was no miasma in the air; on the contrary, I fully believe there was; but it was not very active till the rains came and the dew point rose to 80 or 90, thus preventing a salutary perspiration, shutting out oxygen, and depressing the whole vitality. When I spoke of the tremendous rains of Africa as followed by sickness, I did not mean that water from clouds was the direct cause thereof, but that it merely afforded the material of vapor. It rains and shines in quick succession and the whole country is therefore a vapor bath.

Some people have no conception of moisture which they cannot see and feel and shake from their clothes; this seems to be the Reviewer's fate thro' all that he has written; but my reasoning supposes water dissolved in the air and not to be seen, felt or ascertained except by the hygrometer or by its depressing influence on the body and mind. I spoke of heavy rains, but merely as affording the hypostasis of vapor. It is this and not fogs that Dr. Willis, Mr. Hopkins, and our own illustrious Dr. John Bell have supposed was the great remote cause of malarious fevers. But the Reviewer begins his attack upon me with four separate fogs—the

East, the West, the South, and a certain other fog that dear good Dr. Wistar used to speak of; and he maintains that they are not morbific. He must mean that they do not cause malarious fevers, for he can think of nothing else. Thus far he is certainly right; but if he means that they are never the remote or the exciting causes of inflammatory disease, he is as certainly wrong. See professor Bell's paper quoted in my discourse.

But why name fogs, a word that is not to be found in my whole paper? When vapor is reduced to fog, it is like rain, comparatively innocuous in summer. One might suppose that the Reviewer had lived in Bœotia or Abdera where fogs hebetate the mind; but as he does not appear to be deficient in other compositions, and moreover, since by turning to his title page you find him most honorably allied, you may rather infer that his dullness was temporary and that he wrote this review during the prevalence of a south and vaporous wind, the "*plumbeus Auster.*" You would not believe that, unless under some sinister influence, a man of his gravity would practise the art of punning which he well knows is the lowest species of wit and never to be used in a grave subject. He calls the paper he criticises *hydrophobous* and like a child, "pleased with a rattle,tickled with a straw," he repeats the witty epithet, I shall not stop to inquire how many times. How he would despise me were I to call his writing *tintinnabulous*, in allusion to his name!

Had he not "*set about his review with a confused set of ideas*" in a vaporous air, he would have clearly seen that I did not deprecate an abundance of water in our streets, but the prevalence of invisible vapor, and he would have called my paper—*steam-fearing*. For I said, let the water pour down our gutters in the greatest abundance, as it passes on rapidly in a narrow stream and has not time to evaporate;" and I direct that the scavengers shall sprinkle before they sweep and scrape, because "this lays the dust which is raked into heaps and hauled away before there is time to do mischief." And yet he has the hardihood to say that I recommend a broom "in place of the sprinkling of water," when in truth I urge both broom and sprinkling in the same sentence!

There is one thing, however which I neglected to say—that tho' light showers and mere sprinkling are injurious, heavy washing rains in a paved city must be salutary; and there is another thing which I said unjustly—I called the Schuylkill water *pestiferous*.

This was certainly a word too strong, but surely I did not mean that it contained the seeds of the plague, or any other specific disease, merely that it was fraught with foreign matters inimical to health. I ought not to have used this word, but in the hurry of composition some unguarded expression must often escape. Something too must be conceded to *hyperbole* a figure that pervades the best of writing—St. John says that if all the deeds of the Saviour were recorded, the world would not contain the books.

Let us say a few words about the wonders of Louisville. He says " that before the streets of that city were paved, the rains of summer caused a great mortality. This was not on account of the mere humidity of the atmosphere, for the rains that fall in summer now, make the air as humid as that of 1822, but without any evil effect." Whether the air of that city is as humid *now*, which means of *late years*, as it was in 1822, may be judged of by what the lamented Professor John. P. Harrison says, *Phil. Jour.* vol. viii. p. 8. " In the opening of 1822, we had an almost unprecedented quantity of rain ; and during June, July, August, and September, an unusual succession of heavy and continued showers. The rain though it fell in excessive abundance and in heavy torrents, seemed to have no effect in cooling the atmosphere. There was a coolness and sultriness proving very oppressive during the summer months, which with little or no abatement continued through the night as well as day." And Prof. Yandell says, *Trans. of the Amer. Med. Ass.* vol. ii. p. 612, that Louisville was formerly called " the grave yard of the West" but that now it is esteemed one of the most healthy cities: that it was subject to bilious fevers that rivalled yellow fever in malignity : that the most fatal of these epidemics broke out in 1822 after a hot rainy season: that at this time only one street was paved and there were eight ponds within the limits of the city most of which were dried up in the course of the summer, exposing foul bottoms to the sun.

Now is it possible under all these circumstances of inordinate rains and heats and ponds and unpaved streets covered with mud, that the air was not more humid than at present ? or does the Reviewer mean that the sprinkling machines supply the place of ponds and mud and African rains ?

Let us here step aside to observe that in 1819 and 20, the people of Louisville enjoyed the benefits of dryness. The summer and fall of these years, says Dr. Harrison, *Phil. Jour.* vol. viii, p. 7. "were remarkably healthy—they were very dry—but some of the weather was as warm as that of the subsequent years. In each of these years little rain had fallen in the spring and early summer months. The ponds in and near the town were evaporated and quite free of water before the first of July in each year. The river was low during both the summers of the above years and there was deposited on the shores and rocks near the town a considerable quantity of drift wood and other matter left by the subsidence of the river and creek." See here the happy effect of dryness, which prevented the action of the febrifacient materials left in the ponds, on the streets, and on the banks of the river. *The next two years were rainy and sickly.*

It might be inferred from the Reviewer that Louisville has more need of sprinkling than most other cities. He says—"it is impossible that arid streets and an atmosphere loaded with impalpable dust of carbonate of lime, can be salubrious." There is no such dust in Philadelphia for which alone I wrote, and therefore, what doleful effects it might produce, I cannot say; but much other dust is borne by the lungs without serious injury. Colliers and all who work in clouds of coal dust are very healthy; millers are healthy except their peculiar cough; in Egypt the blowing sand is so abundant as to keep almost every one's eyes sore, yet it inflicts no other sensible injury; the caravans on the deserts of Asia and Africa, are not much injured in their health by storms of sand, although these are sometimes so thick as to kill suddenly by shutting out oxygen; the clouds of thick dust that attend the marching of armies day after day are not very serious evils. That dust is a little annoying, is freely conceded; and from the Reviewer's horror and his desire to sprinkle it down, one might infer that he was as restive as other people under the yoke of Providence. I have been told that Cincinnati is paved with a friable carbonate of lime and that the dust is often intolerable; if Louisville is thus paved, let them sprinkle as the choice of two evils.

That some kinds of dust as that of dry-grinders and pearl-workers, is injurious, has been sufficiently proved; but I cannot find that the citizens of Phila. are set to coughing by the inhalation of street dust. Nine-tenths of the time, it is not even perceived. Our citizens in riding abroad for their pleasure or health, pass each other by crowds in Broad Street beyond the pavement where they are covered with dust, preferring this to the damp and confined air of the city, where dust is comparatively very rare. I cannot remember that in walking or driving in the streets of Philadelphia I have been annoyed by dust this summer, yet this is the middle of August and not a mile of the city is sprinkled by the machine. An old man who has been in the constant practice of giving advice in relation to health, will often be obtrusive, hence I cannot help saying to this Louisville Doctor—clean your streets before you sprinkle them. What *in loco cit.* says Dr. Yandell the presiding genius of that city? "It would certainly be difficult to find any where more uncleanly lanes and thoroughfares than Louisville presents. One of the first things that strikes the eye of a stranger is the neglected condition of our streets. He wonders how the city can be healthy with such masses of putrescent matters in its midst." But he adds—"the streets of the city are wide and cross at right angles, which secures the most perfect ventilation and *favors their drying fast after rains.*" This was written only sixteen months ago. The following four short paragraphs contain a surprising number of errors and rash assertions.

"But let us survey some other parts of the earth. One of the healthiest portions of Devonshire, in England, is Torquay. It is a resort for a considerable number of invalids. But the humid state of the atmosphere of Torquay is notorious.

"The south of Italy has long been celebrated for its salubrity, but it is no less famous for its humid air, and Rome, the most humid part of the south of Italy, has the best climate for invalids. Madeira has long been the shrine of Hygeia, to which pilgrims from all parts of the world have resorted, but it, too, has an atmosphere that can scarcely be called dry.

"In the West Indies the hot, dry months are not healthy. The invalid is urged to spend all the time he can upon the water, where the atmosphere is much more humid than that of the land. A ship may be 'dry' but its atmosphere is not.

"An important fact should also be borne in mind, and that is, that there are many diseases which are benefitted by humid air, while there are others injured by it. While, therefore, Dr. Jackson's hydrophobia might benefit one set of invalids, it would be deleterious to another."

The truth is that Devonshire is the south of England, on the channel bordering France, and of course the air is comparatively warm; hence a number of situations therein have been sought for a winter residence—not because the air was supersalubrious, but because it was warm for an English or Scotchman. It is convenient for those Northern men who cannot leave the island, but did you ever hear of invalids going there from neighboring France or any other foreign land? The Reviewer says " the humid state of Torquay is notorious." Sir James Clarke, the highest authority, says otherwise—hear him.

" Here the invalid has the advantage of a considerable tract of sheltered country, some part of which will afford a protected ride or walk in whatever direction the wind blows. Torquay is superior in this respect to every place in our island. Its position also on the southern declivity of a range of pretty steep hills, composed chiefly of calcareous rocks, renders it *comparatively dry*. Hence while Torquay possesses all the advantages of the climate of this coast, its chief disadvantage, *humidity*, is felt in a less degree than elsewhere."—*Cyclop. of Pract. Med., Art. Climate.*

As to the south of Italy, did any one ever hear of its salubrity? Consumptive, rheumatic, and bronchial patients may go there to spend a winter, but this is all that can be said in its favor. So they go to the West Indies, but no father or mother would go to either place to rear a robust family, and this is the very thing that my steam-fearing relates to. He says that " Rome is the most humid part of the south of Italy." For this he ought to have given some proof. Sir James Clarke says it is dryer than Pisa which is nearly two degrees further north, and yet on the southern coast. In the country south of the Appenines and below the latitude of Rome down to the toe of the boot, you may find many places more humid than Rome. It is at Naples and still further to the south-east, that the moist sirocco blows for weeks, relaxing every fibre and dejecting every mental energy. Whoever would say " the south of Italy has long been celebrated for its salubrity," would do well to read Dr. Jas. Johnson on " Change of Air " and the Rev. Mr. Mathews' Diary.

He says that in the West Indies " the hot dry months are not healthy." Let him say whether the hot *wet* months are healthy. Neither dry nor wet months are healthy in this region unless you reside in the mountains. If the dry hot months should at any time prove the least healthy, it is because they indirectly favor the generation of malaria and this I mentioned in my preface; but look into Hillary on "Change of Air," you will find that July and August were unhealthy in proportion to the quantity of rain; and Mosely begins his summary of advice to Europeans arriving in the West Indies thus—"Living in a house with lofty and spacious rooms in a *dry* situation, &c." As to people seeking their safety on the water, this is merely the less of two evils, for surely no one is crazy enough to pretend that a ship in the torrid zone is a healthy situation. The inmates may not contract the specific fever of the neighboring land, they may not die, but they will be broken down in their vigor and elasticity.

He says that Madeira is the shrine of Hygeia and that "it can scarcely be called dry." Suppose I too were to guess and rashly say, it can scarcely be called humid. The pure air from the sea blows over it and carries away its impurities; and during the hottest weather, the sun being in his extreme northern declination, the island is under the purifying influence of the trade winds. The island rises rapidly from the coast by a succession of lofty hills, the soil is desiccative and the streams very rapid. For six and often for eight months, the silk worm might be fed on the trees, for during that period it seldom rains except in the mountains.—*See Rees' Cyclop.*

Dr. Gourlay, who had resided there eighteen years when he wrote, says that "a drop of dew seldom falls except in the higher parts:" and Dr. Joseph Adams then residing on the island, (*Med. and Phys. Jour. for* 1801,) speaking of the charming temperature says, "the dryness of our atmosphere is not less remarkable." Then he speaks of the absence of fogs and the rapidity of rivers. Dr. Wilde, *p.* 89, says the "dry and balmy air which produces this never-ending spring, &c." At *p.* 113 he says, "with so little rain and dew, it may naturally be asked, how vegetation can be so luxuriant"—he means in and near Funchal, where the foreign invalids reside. The above will help to show that the Reviewer's *salutary* fogs are not to be expected here and that the island can scarcely be called humid during six or eight months. But he says it is the "shrine of Hygeia." Dr. Gourlay thinks otherwise and says *Part III.*, it is equally the seat of disease as other countries, and the forms of these are neither few in number nor mild in their nature."

But with respect to all these moist places mentioned triumphantly by the Reviewer—Torquay, Southern Italy, West Indies, Madeira—not one of them is rendered useful to invalids by its humidity, or recommended to the sick on account of this property; there is not one of them to which a physician in his right mind would resort for the purpose of rearing a family in health, longevity, and vigor. Invalids and aged people run there in winter to escape frost and chimoniphorous mountain winds, just as they would cover their belly with pitch to prevent cholera. These places, they think, have the desirable uniformity of temperature, and their humidity *during the winter*, is not as injurious to them as the evils they fly from. Thus some physicians in England a few years ago, sent patients to pass the cold weather in cow-houses, in order to breathe day and night the milder air from their cattles' lungs and bowels, and I believe it is practised in France to this day; but did the same doctors advise the whole world sick and well to do the same?

To his "important fact" that many diseases are benefitted by humid air, I have only to reply, there is not one in the earth that could be relieved by air more humid than ours of Philadelphia generally is in the summer months. Here too, in allusion no doubt to my story of Capt. Murray's dry ships, he says " a ship may be dry but its atmosphere is not." O, wonderful! But may not the internal atmosphere of a ship be rendered drier by such means as Capt. Murray used?

The Reviewer says "it seems to us that Dr. J. set about his investigation with a confused set of ideas and that the confusion increased greatly towards the close of his paper." This assertion he no where attempts to prove unless at p. 11, where he writes as follows—

" We have remarked that Dr. Jackson's confusion increases towards the conclusion of his essay. He finds it difficult to meet the objection urged against his views, in the appeal to the genial influence of showers of rain. He solves the difficulty by declaring that there is no kind of comparison between 'pure water from the higher regions, which in its descent washes and purifies the air,' and 'the impure and pestiferous water of the Schuylkill.' This then is a surrender of the whole question. If Dr. Jackson had started out with the proposition that sprinkling streets with impure and pestiferous water is injurious to health, he would have found no one to controvert his position. Truth forces him to admit that streets may be sprinkled with pure water, without any injurious effect.

"But there is another important admission in Dr. Jackson's effort to meet the fact we have mentioned, an admission that is fatal to his entire series of speculations. In announcing that falling rain washes out impurities from the atmosphere, he admits that a dry air may be unwholesome. These impurities spring from the bosom of the earth, and nine-tenths of them are of a character that may be kept from a breathing atmosphere, by sprinkling the streets with pure water. The hydrants and fountains of New York and Boston, are not detrimental to health."

My whole reasoning goes upon the fact, that an inordinate quantity of water *pure or impure* dissolved in the air as it is during a south wind, depresses the vital powers and is therefore more or less injurious to health, independently of its favoring the remote causes of disease. But as I was writing for this city in particular, I spoke of the impurity of our water as an *accumulative* evidence, to use a law phrase, against the sprinkling, precisely as I spoke of miasma-gennetic materials in the streets; I have no where admitted, as he asserts, that streets may be sprinkled with pure water without any injurious effects. No such admission can be inferred from any part of my paper; not from the case of Capt. Murray's ship, Blagden and Fordyce's experiments, Willis's theory, the long extract from Dr. John Bell, nor yet from any thing in the deaths at the battle of Monmouth. I set out at the first with this proposition—"that a great excess of heat is tolerated by the human body without injury, provided there be no excess of moisture," and to this proposition I have steadily adhered. He says "Truth forces him to admit that streets may be sprinkled with pure water without any injurious effects." This is another *constructive* accusation and I disdain to answer it; but the Reviewer might be advised to remember how Peter found what he wanted in his father's will—in syllables and letters.

The Reviewer slyly observes that the hydrants and fountains of New York and Boston are not injurious to health. How could they possibly injure health on my principles? They are not calculated to afford an invisible steam. The water pours up cold to a certain height, then falls into a basin and runs off under ground. It has not time to absorb a vaporising heat; and the little caloric that must be combined, is quickly carried under the earth. Some little vapor must be produced, but not enough to do any evil. The same may be said of the hydrants that wash the gutters.

As to the " important admission which is fatal to *my* entire series of speculations that dry air may be unwholesome and that rain washes out its impurities," it is only necessary to say, that all air, dry or moist, is more or less impure particularly in cities ; and I freely admit as I did in my paper that rains do wash and purify it. But I ought to have said *heavy* rains, for light showers that merely lay the dust, as does the sprinkling machine, cannot do this ; on the contrary, they never can fail to increase the impurity of the air—they promote putrefaction and exhalation which rises around us and is precipitated within reach of our lungs by the dew, to rise again with the next day's heat and vapor. This too must be the effect of sprinkling the streets to any great extent even with the purest water.

That very great man, Dr. Rush, used to teach us that light showers were always injurious in a sickly season. But the Reviewer says " impurities spring from the bosom of the earth and nine-tenths of them may be kept from a breathing atmosphere by sprinkling the streets with water." I prefer the authority of Dr. Rush. But if this need any support, let us see what some of the wise and learned physicians of Philadelphia have said on the subject. Among many questions proposed by the Councils to these men individually, is found the following :—

" Is it proper to water or sprinkle the streets before cleaning them or at any other times ; and what precautions if any would it be desirable to employ while cleaning the streets and gutters ?" To this three physicians answered in favor of sprinkling and the following nine against it.

Professor Dunglison says—" The undersigned can have no facts to offer. Believing however as he does that the hygrometric condition of the air is much concerned in the production and extension of disease, he would be disposed to prefer the dry cleaning of the streets to the moist." He recommends the flowing of water through the gutters night and day.

Professor Bache says—" It is proper to water or sprinkle the streets before cleaning them, if they are dry or dusty ; but not at other times."

Professor Gibson—" The streets should not be *sprinkled*, but thoroughly cleaned by opening fire plugs and hydrants and washing away with a deluge of water all foul matters. Desiccation follows sprinkling and this is believed to be deleterious."

Dr. J. W. Moore—"I do not think there is any objection to sprinkling the streets for the purpose of cleansing them; but I do think the practice of watering them in hot weather prejudicial."

Professor Meigs—"The use of hose to water the streets at the discretion of citizens and servants, ought to be forbidden, inasmuch as in many of the principal thoroughfares, the dust is constantly kept in a state to favor the rapid decomposition of matters mixed with it, giving rise *not only to unwholesome damp* but disengaging chemical vapours that are injurious to health. If we should be subject to cholera, we should be safer in a dry than in a damp city. Our city is too much watered at seasons in which excessive moisture ought to be avoided. As to cleansing the streets, I advise that they be swept while dry.

Professor Mütter—"As moisture when combined with heat is one of the chief sources of malaria, it must be obvious that to sprinkle a dirty street or alley and allow the surface to dry by degrees, cannot fail to be injurious to health. This however is very different from a thorough washing, and I advise that the hydrants be allowed to flow every day or two. Where no hydrants exist, I believe dry sweeping will be better than sprinkling."

Professor Sam. Jackson—"In dry weather sprinkling or wetting the streets before sweeping is required to lay the dust; but the dust should not be converted into mud which defeats the object of sweeping."

Professor Grant—"I consider the practice of watering the streets during the heat of the day for the purpose of laying the dust &c. as very objectionable. Every such sprinkling promotes decomposition. I suggest that the citizens should not be permitted to sprinkle the streets &c."

Dr. Isaac Hays—"It is proper in very dry weather to sprinkle the streets before sweeping them; but it cannot be conducive to health to keep the streets constantly sloppy."

While I have such men as these on my side and many others who ought to be named, I shall feel as confident as did a great actress of old whom the vulgar exploded and the knights applauded.

> ——nam satis est *equites* mihi plaudere, ut audax
> Contemptis aliis explosa Arbuscula dixit.

We shall not quote the next lines, sincerely believing they are not suitable; for notwithstanding the apparent temper of the Reviewer, we believe that its author is a man like Sir Jonas Hanway, " whose vices may well be forgiven for the sake of his virtues." He has shown some cloudiness of mind, the temporary effect no doubt of vapor from the sprinkling machine ; and a spice of ill-nature, which may be imputed to a bad state of the bowels, as indigestion and flatus, says Voltaire, in ministers of state, occasion nearly all the wars of Europe. He seems to be a *religiosissimus* and very irritable on the subject of religion ; now as a few barrels of oil have been said to smooth down the angry waves, it might be well for him to take some cod liver.

I have now, Mr. Editor, spent too much time on this subject and therefore I bid it a final farewell.

